# Nanopore-Based Comparative Transcriptome Analysis Reveals the Potential Mechanism of High-Temperature Tolerance in Cotton (*Gossypium hirsutum* L.)

**DOI:** 10.3390/plants10112517

**Published:** 2021-11-19

**Authors:** Yajun Liang, Zhaolong Gong, Junduo Wang, Juyun Zheng, Yizan Ma, Ling Min, Qin Chen, Zhiqiang Li, Yanying Qu, Quanjia Chen, Xueyuan Li

**Affiliations:** 1 Engineering Research Centre of Cotton of Ministry of Education, Xinjiang Agricultural University, 311 Nongda East Road, Urumqi 830001, China; 13999966149@163.com (Y.L.); cqq0707@sina.com (Q.C.); xjyyq5322@126.com (Y.Q.); 2 Xinjiang Academy of Agricultural Science, Urumqi 830001, China; g15981775091@163.com (Z.G.); 13579975299@163.com (J.W.); zjypp8866@126.com (J.Z.); 3 National Key Laboratory of Crop Genetic Improvement, Huazhong Agricultural University, Wuhan 430070, China; mayizan@webmail.hzau.edu.cn (Y.M.); lingmin@mail.hzau.edu.cn (L.M.); 4 Adsen Biotechnology Co., Ltd., Urumqi 830022, China; lizq19900125@163.com

**Keywords:** cotton, high-temperature, transcriptome, tolerance

## Abstract

Extreme high temperatures are threatening cotton production around the world due to the intensification of global warming. To cope with high-temperature stress, heat-tolerant cotton cultivars have been bred, but the heat-tolerant mechanism remains unclear. This study selected heat-tolerant (‘Xinluzao36′) and heat-sensitive (‘Che61-72′) cultivars of cotton treated with high-temperature stress as plant materials and performed comparative nanopore sequencing transcriptome analysis to reveal the potential heat-tolerant mechanism of cotton. Results showed that 120,605 nonredundant sequences were generated from the raw reads, and 78,601 genes were annotated. Differentially expressed gene (DEG) analysis showed that a total of 19,600 DEGs were screened; the DEGs involved in the ribosome, heat shock proteins, auxin and ethylene signaling transduction, and photosynthesis pathways may be attributed to the heat tolerance of the heat-tolerant cotton cultivar. This study also predicted a total of 5118 long non-coding RNAs (lncRNAs)and 24,462 corresponding target genes. Analysis of the target genes revealed that the expression of some ribosomal, heat shock, auxin and ethylene signaling transduction-related and photosynthetic proteins may be regulated by lncRNAs and further participate in the heat tolerance of cotton. This study deepens our understandings of the heat tolerance of cotton.

## 1. Introduction

Cotton (*Gossypium hirsutum* L.), an essential industrial and economic crop, is widely cultivated across the world. As the world population grows and industrialization advances, global warming accelerates, resulting in a rise in extreme weather events such as high temperatures, flood, and drought [1,2]. High temperatures are one of the most serious risks to agricultural productivity because they directly impact plant development via biochemical activity in plant cells [3]. Cotton is a thermophilic crop that depends on high temperatures to ripen. The optimum temperature range for cotton growth is 28–35 °C, with temperatures exceeding 35 °C considered exceptionally high and affecting cotton growth and development [4,5]. However, cotton, by comparison, frequently suffers from extremely high temperatures during summer flowering [6]. Extremely high temperatures have a negative impact on cotton growth, resulting in a hindered reproductive process, difficult dehiscence of anthers, and lower pollen activity [4].

To develop more high-temperature-resistant cotton varieties, some studies have made efforts to uncover the responses to high-temperature stress of cotton at the molecular level. A recent study revealed that microRNAs (miRNAs) are differentially expressed under different temperature treatments in cotton and their target genes are enriched in response to hormone stimulus, photosynthesis, and plant hormone signal transduction pathways [7]. Another study further showed that miRNAs that respond to high-temperature stress are developmental stage-specific in cotton anthers [8]. MiRNAs regulate cotton anther fertility under high-temperature stress by mediating auxin signaling. In particular, overexpression of miRNA156 and miRNA157 decreases high-temperature tolerance in the cotton anther, leading to anther indehiscence [6]. In addition to miRNAs, studies reported many functional genes that may be involved in high-temperature resistance in cotton. Ma et al. (2021) found that a heat-susceptible gene, designated as heat-related receptor kinase *GhHRK1*, negatively responds to high-temperature stress in cotton, whereas the *Arabidopsis* homologous *GhHRK1* mutant shows strong high-temperature tolerance, indicating that this gene may play a key role in high-temperature resistance in cotton. Some genes related to cytokinin, abscisic acid, and brassinosteroid signal transduction, such as *CRE1*, *ABF*, and *CYCD*, are considered to participate in the regulation of high-temperature response in cotton, thus contributing to the maintenance of plant growth [9]. Heat shock reaction is a common physiological phenomenon that widely exists in biological cells. In this kind of biological reaction, the synthesis of heat shock protein (HSP) is closely related to the acquisition of heat resistance of organisms [10]. HSPs provide a protective mechanism for cells to resist high-temperature stress. Under high-temperature stress, plants produce HSPs to protect body proteins from damage or repair damaged proteins, thereby playing a protective role for plants [11]. These findings help us better understand the molecular responses of cotton to heat stress, but they do not fully reflect the heat-resistance mechanism of cotton.

As a result of the development of sequencing technology, third-generation sequencing, namely, Pacific Biosciences (PacBio) and Oxford Nanopore Technologies (ONT), has been increasingly utilized in biological science research. They provide huge advantages in sequence length, transcript identification, and genetic information richness compared with second-generation Illumina RNA-seq technology [12,13]. Furthermore, a comparative study between PacBio and ONT transcriptomic sequencing of *Arabidopsis* showed that they have similar efficiency in transcript identification and long non-coding RNA (lncRNA) prediction, but ONT costs less and can quantify expression levels of transcripts compared with PacBio sequencing [14]. ONT sequencing was recently used for the scientific study of several plant species, such as *Gnetum luofuense* [15,16] and sunflower [17]. A transcriptome study of *Camellia sinensis* cv. Fudingdabai showed that the gene expression levels generated from the Nanopore and Illumina sequencing were coincident [18]. Moreover, ONT has been used for genome assembly of circum-basmati rice and showed high efficiency [19]. The results of these studies provide us with abundant molecular information on these species. However, ONT sequencing has not been widely applied in phytobiological research.

In the present study, a heat-tolerant cultivar and a heat-sensitive cotton cultivar were selected as the plant materials. The two cultivars were treated with a heat-stressed temperature, and the leaf samples were collected for ONT sequencing. The transcriptome of the two cultivars was comparatively analyzed to reveal the potential heat tolerance mechanism of cotton. The results of this study will deepen our understanding of heat resistance in cotton and provide candidate genes for the genetic breeding of cotton.

## 2. Results

### 2.1. Overview of ONT Sequencing

A total of 24 libraries that represented three biological replicates of the eight groups of samples were generated from ONT sequencing. Clean data were obtained from the raw data by filtering the short and low-quality reads. The average and N50 lengths were distributed at 882–1057 and 927–1153 bp of the clean data in each library, respectively (Supplementary Table S2). The Q score of the clean data was 11, indicating that the libraries had good quality. The full-length percentage in each library was distributed from 76.77% to 81.39% (Supplementary Table S3). The full-length reads were further clustered and mapped to the reference genome to remove redundancy, generating 120,605 nonredundant sequences.

**Figure 1:**
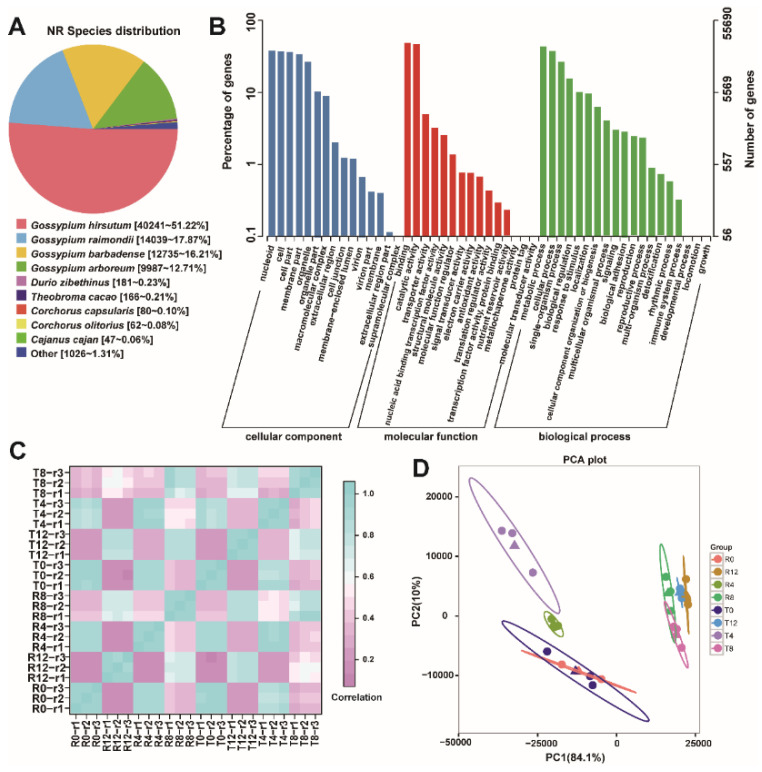
Overview of the ONT sequencing results. (**A**) Homogeneous analysis of the Nr species distribution; (**B**) GO term statistics of the annotated genes; (**C**) correlation between the samples; (**D**) principal component analysis of the samples.

The nonredundant sequences were annotated from the eight databases and generated a total of 78,601 genes. Annotation results showed that 55,690, 26,860, and 78,569 genes were annotated from the GO, KEGG, and Nr databases, respectively (Table 1). Analysis of Nr homologous species distribution showed that the annotated genes in the current study were most similar to those from *G. hirsutum* (51.22%), *Gossypium raimondii* (17.87%), and *Gossypium barbadense* (16.21%) (Figure 1A). GO enrichment analysis of the annotated genes revealed that the most enriched GO terms were nucleoid, cell, cell part, and membrane part in the cellular component; binding and catalytic activity in molecular function; metabolic process and cellular process in the biological process (Figure 1B). The repeatability of the samples was evaluated by calculating Pearson’s correlation coefficient between the samples according to the expression levels. As shown in Figure 1C, samples showed high intra-group correlations (0.91–1.00), indicating good biological repeatability of the samples. Principle component analysis revealed that the samples in different treatment groups exhibited a discrete pattern (Figure 1D), especially the samples of the tolerant type, indicating that high-temperature treatment posed a remarkable influence on the two cotton types.

ONT sequencing can help identify new genes that do not match the reference genome. Here, a total of 6415 new genes were obtained by annotating from the eight databases (Supplementary Table S4). Nr homologous species analysis of the new genes showed that they were homologous to the genes from *G. hirsutum* (25.89%), *G. raimondii* (25.54%), and *G. barbadense* (22.61%) (Supplementary Figure S1A). GO enrichment analysis showed that the new genes were enriched in membrane, cell, cell part, and membrane part of the cellular component; binding and catalytic activity of molecular function; and metabolic process and cellular process of the biological process (Supplementary Figure S1B). The expression profiles of the new genes showed that they exhibited different expression patterns under varying harvest times after high-temperature treatment (Supplementary Figure S1C).

### 2.2. Enrichment Analysis of All the DEGs

**Figure 2:**
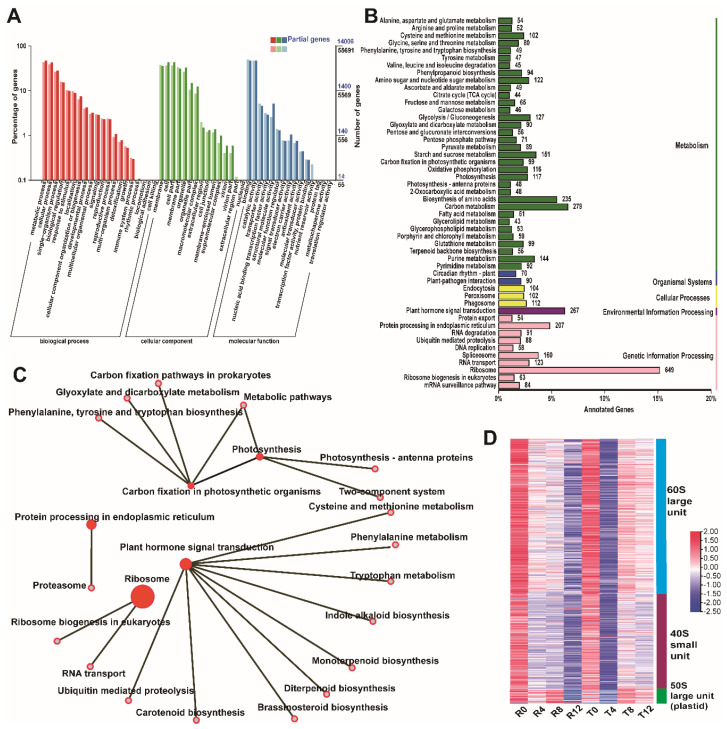
Analysis of all the DEGs. (**A**) GO enrichment analysis; (**B**) KEGG pathway enrichment analysis; (**C**) relationship between the KEGG pathways; (**D**) expression profile of the differentially expressed ribosomal protein genes.

We established 14 comparison groups to screen DEGs: R0 vs. R4, R0 vs. R8, R0 vs. R12, R4 vs. R8, R8 vs. R12, T0 vs. T4, T0 vs. T8, T0 vs. T12, T4 vs. T8, T8 vs. T12, R0 vs. T0, R4 vs. T4, R8 vs. T8, and R12 vs. T12. A total of 19,600 DEGs were screened from the 14 comparison groups (Supplementary Table S5). The expression heatmap of all the DEGs indicated that they had different expression patterns in various samples (Supplementary Figure S2). These DEGs were conducted with GO term and KEGG pathway enrichment analyses. GO term enrichment analysis showed that 14,006 DEGs were annotated in the GO database. In the cellular component cluster, the largest sub-categories were membrane, cell, cell part, membrane part, and organelle, and they contained 4986 (35.60%), 6103 (43.57%), 6009 (42.90%), 4346 (31.03%), and 4562 (32.57%) genes, respectively. Binding and catalytic activity were the two most enriched GO terms in molecular function, and they accounted for 6552 (46.78%) and 6492 (46.35%) of all the GO annotated DEGs, respectively. In the biological process category, metabolic process, cellular process, and single-organism process were the top sub-categories, which accounted for 6559 (46.83%), 5978 (42.68%), and 3948 (24.97%) genes, respectively (Figure 2A). A total of 4283 DEGs were annotated in the KEGG database. The statistics of KEGG pathway enrichment of the DEGs revealed that ribosome (Ko03010), carbon metabolism (Ko01200), plant hormone signal transduction (Ko04075), biosynthesis of amino acids (Ko01230), and protein processing in endoplasmic reticulum (Ko04141) were the top enriched pathways (Figure 2B). To obtain a global picture of the connections between these KEGG pathways, we constructed a network between them and their biological adjacent pathways (Figure 2C). In the network diagram, the solid circle represents the core pathway, whereas the hollow circle represents the adjacent pathway. The size of the solid circle represents the number of enriched genes. Here, the ribosome pathway contained the largest number of DEGs, and it was correlated with RNA transport and ribosome biogenesis in eukaryotic pathways. The protein processing in endoplasmic reticulum pathway was linked to the proteasome pathway. Carbon fixation in photosynthetic organisms, photosynthesis pathways, and their adjacent pathways (e.g., metabolic pathways and photosynthesis-antenna proteins) constituted a network. The plant hormone signal transduction pathway was linked to the largest number of neighbor pathways, such as tryptophan metabolism, diterpenoid biosynthesis, and ubiquitin-mediated proteolysis.

Given that the ribosome was the most significantly enriched pathway, the DEGs involved in this pathway were further analyzed. The results showed that 526 DEGs belonged to the ribosomal protein family, and they were categorized into three clusters, namely, *60S large unit*, *40S small unit*, and *50S large unit (plastid) ribosomal proteins*. Among the ribosomal protein genes, the *60S large unit ribosomal protein* subfamily contained 298 DEGs, which were annotated into 45 members of the *60S ribosomal protein* subfamily, such as *60S ribosomal protein L40*, *L9*, *L18*, and *L26*. The *40S small unit ribosomal protein* subfamily contained 196 DEGs. They were annotated into 34 members of the *40S small unit ribosomal protein* subfamily, including *40S ribosomal protein S10*, *S16*, *S18*, and *S30*. The remaining 32 DEGs were annotated into 19 members of *50S large unit (plastid) ribosomal proteins*, such as *50S ribosomal protein L13*, *L9*, and *L34*. Expression analysis of the DEGs involved in ribosomal protein showed that they remarkably responded to high-temperature stress. As shown in Figure 2D, the expression levels of these DEGs from the sensitive cultivar decreased with the treatment time and were significantly lower in R12 than in R0. However, the *ribosomal proteins* demonstrated significantly decreased expression levels in T4 than in T0, but their expression levels were upregulated with the extension of high-temperature treatment time.

### 2.3. DEG Analysis between the Sensitive and Tolerant Types

**Figure 3:**
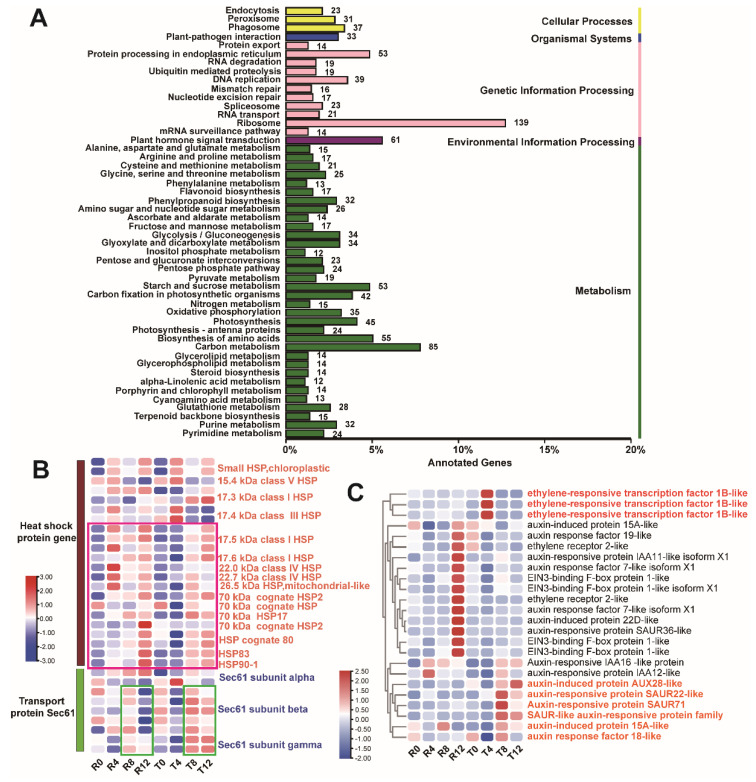
Analysis of the DEGs from the comparison of the two cotton cultivars. (**A**) KEGG pathway enrichment analysis; (**B**) expression profile of the DEGs in the protein processing in endoplasmic reticulum pathway; (**C**) expression profile of the DEGs in the plant hormone signaling transduction pathway.

We screened 5075 DEGs from the comparison between the sensitive and tolerant type including four comparison groups (R0 vs. T0, R4 vs. T4, R8 vs. T8, and R12 vs. T12) and 1093 of them were annotated from the KEGG database. Similar to the results of KEGG pathway enrichment of the entire DEGs, these 1093 DEGs were dominantly concentrated in the ribosome, carbon metabolism, plant hormone signal transduction, and protein processing in endoplasmic reticulum pathways (Figure 3A). Considering the pathway connection between the plant hormone signal transduction and protein processing in endoplasmic reticulum and their neighbor pathways, as described in Figure 2C, we further analyzed the DEGs enriched in these two pathways. Among the 53 DEGs involved in the protein processing in the endoplasmic reticulum pathway, 22 of them were annotated as *HSP*, and nine of them were annotated as *transport protein Sec61* (*Sec61*). The 22 DEGs were annotated into 17 *HSP* members, such as *17.5 KDa class I. HSP* (*HSP17.5*) and *70 KDa HSP* (*HSP70*). Expression analysis showed that 12 *HSPs* (e.g., *HSP17.5*, *HSP70-17*, and *HSP83*) exhibited a similar expression pattern, which enhanced expression levels in R4, R8, and R12 compared with R0, but insignificantly changed in T4 and remarkably increased in T8 and T12 when compared with T0 (Figure 3B). These results reflect the difference between the sensitive and tolerant types in the response of *HSP* to high-temperature. Additionally, the expression of *Sec61 subunit beta* and *gamma* showed differences between the two cultivars; the expression levels were enhanced in T8 and T12 but decreased in R12 (Figure 3B).

The DEGs enriched in the plant hormone signaling transduction pathway were mainly involved in auxin and ethylene responses. These DEGs were divided into clusters, which represented three expression patterns (Figure 3C). In the first cluster, the expression level of *ethylene-responsive transcription factor 1B* was significantly higher in T4 than in other groups. The genes in the second cluster, such as *EIN3-binding F-box protein 1-like* and *ethylene receptor 2-like*, presented the highest expression level in R12. The third cluster contained six auxin-responsive protein genes (e.g., *auxin-induced protein AUX28-like* and *auxin response factor 18-like*), which demonstrated significantly enhanced expression in T8 and T12.

### 2.4. Differential Analysis of DEG Enrichment in the Sensitive and Tolerant Types

**Figure 4:**
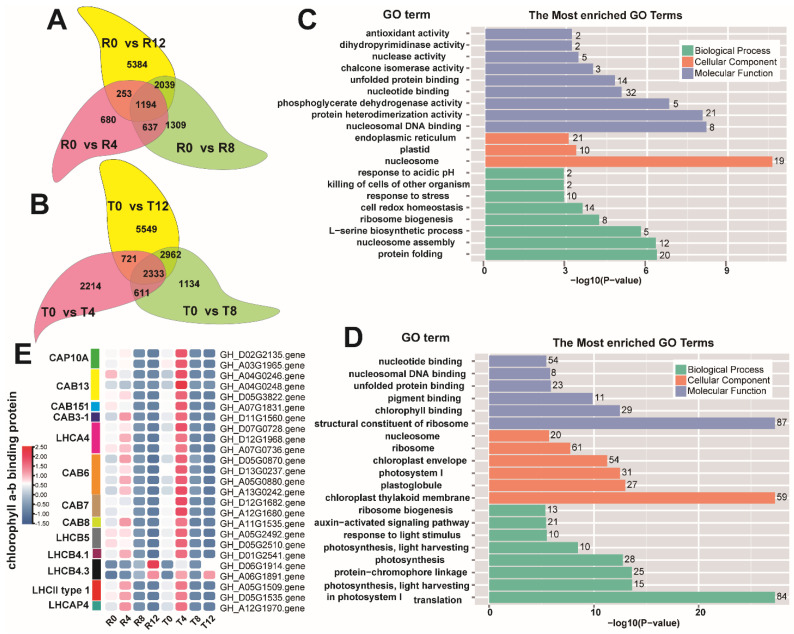
Analysis of the DEGs from the intra-groups of the two cotton cultivars. (**A**) Venn diagram showing the DEG numbers in each comparison group of the sensitive cultivar; (**B**) Venn diagram showed the DEG numbers in each comparison group of the tolerant cultivar; (**C**) the top 20 GO terms of the overlap DEGs in the sensitive cultivar; (**D**) the top 20 GO terms of the overlap DEGs in the tolerant cultivar; (**E**) expression profile of the *chlorophyll a-b binding protein* genes.

To further explore the difference in the DEGs between the high-temperature sensitive and tolerant cultivars, intra-cultivar DEG enrichment analysis was performed. For the sensitive cultivar, 1194 DEGs were found to constitute the overlap of the comparison groups R0 vs. R4, R0 vs. R8, and R0 vs. R12 (Figure 4A). For the tolerant cultivar, 2333 DEGs were found to form the overlap of the comparison groups T0 vs. T4, T0 vs. T8, and T0 vs. T12 (Figure 4B). These overlapped DEGs from the two cotton cultivars were subjected to KEGG pathway and GO enrichment analyses. KEGG pathway enrichment analysis showed that the two cultivars exhibited strong similarity to the overlapped DEGs enriched in the ribosome, protein processing in endoplasmic reticulum, and plant hormone signaling transduction (Supplementary Figure S3). However, analysis of the top 20 GO terms revealed that the overlapped DEGs from the sensitive cultivar were predominantly enriched in the nucleosome, protein folding, and nucleosomal DNA binding (Figure 4C), but the chloroplast thylakoid membrane, structural constituent of ribosome, and translation were the most enriched GO terms of the overlapped DEGs from the tolerant cultivar (Figure 4D). In contrast to the sensitive type, the terms related to photosynthesis, including photosynthesis, light harvesting in photosystem I, photosynthesis, light-harvesting, and photosystem I, were found to be significantly enriched in the tolerant cultivar (Figure 4D). The DEGs involved in these photosynthesis-related terms were screened and found to mainly be *chlorophyll a-b binding proteins*, such as *CAP10A*, *CAB6*, and *LHCB5*. Expression analysis of these genes showed that the most noticeable difference between the two cultivars was the significant increase in their expression in T4.

### 2.5. LncRNA and Target Gene Prediction

**Figure 5:**
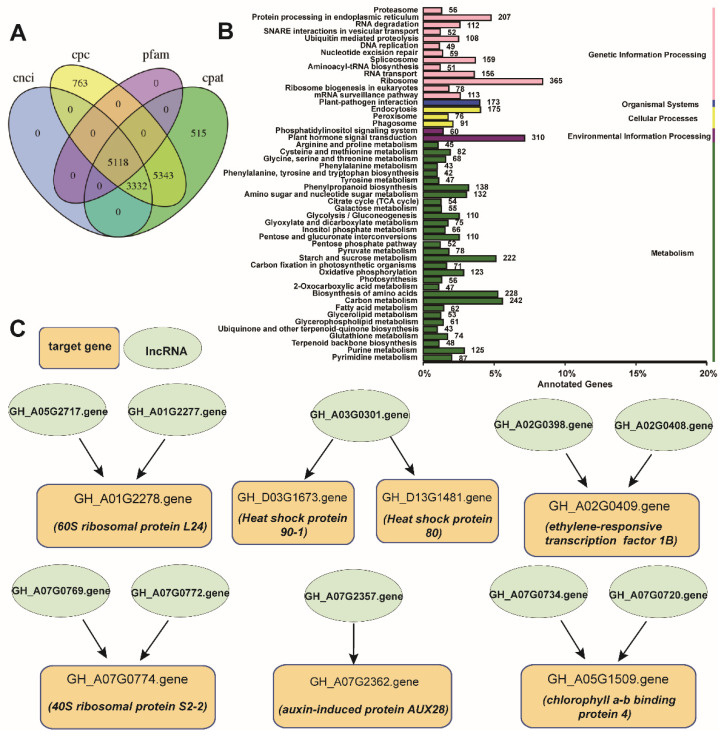
Analysis of the predicted lncRNAs and their target genes. (**A**) Statistics of the lncRNAs; (**B**) KEGG pathway enrichment analysis of the target genes; (**C**) the representative lncRNAs and their target genes.

A total of 5118 lncRNAs were predicted using the four methods: CNCI, CPC, Pfam, and CPAT (Figure 5A). These lncRNAs consisted of four categories, namely, intergenic, antisense, intronic, and sense- lncRNAs, but intergenic-lncRNA was the main form, which accounted for 87.1% (4457) of the lncRNAs (Supplementary Figure S4A). Expression profile analysis of the predicted lncRNAs revealed that most of the lncRNAs exhibited a higher expression pattern at 8 and 12 h under high-temperature treatment compared with those at 0 and 4 h (Supplementary Figure S4B), implying that they may respond to a high temperature in cotton.

The present study also predicted the target genes of the predicted lncRNAs, and a total of 24,462 target genes were obtained. Among them, a total of 17,087 and 4337 were annotated from the GO and KEGG databases, respectively. GO term statistics of the target genes showed that metabolic process, cellular process, membrane, cell, binding, and catalytic activity were the most enriched (Supplementary Figure S4C). KEGG pathway enrichment analysis of the target genes showed that the dominant pathways were ribosome, plant hormone signaling transduction, carbon metabolism, and protein processing in endoplasmic reticulum (Figure 5B). These results were consistent with the results of DEG analysis. We screened several representative target genes and their corresponding lncRNAs to show their relationship. As shown in Figure 5C, seven genes were the target genes of lncRNAs; for example, *60S ribosomal protein L24* was the target gene of lncRNAs GH_A05G2717.gene and GH_A01G2277.gene, whereas HSP90-1 and HSP80 were the targets of the lncRNA GH_A03G0301.gene. These target genes were the representatives of the enriched KEGG pathways.

### 2.6. WGCNA of the Screened DEGs

**Figure 6:**
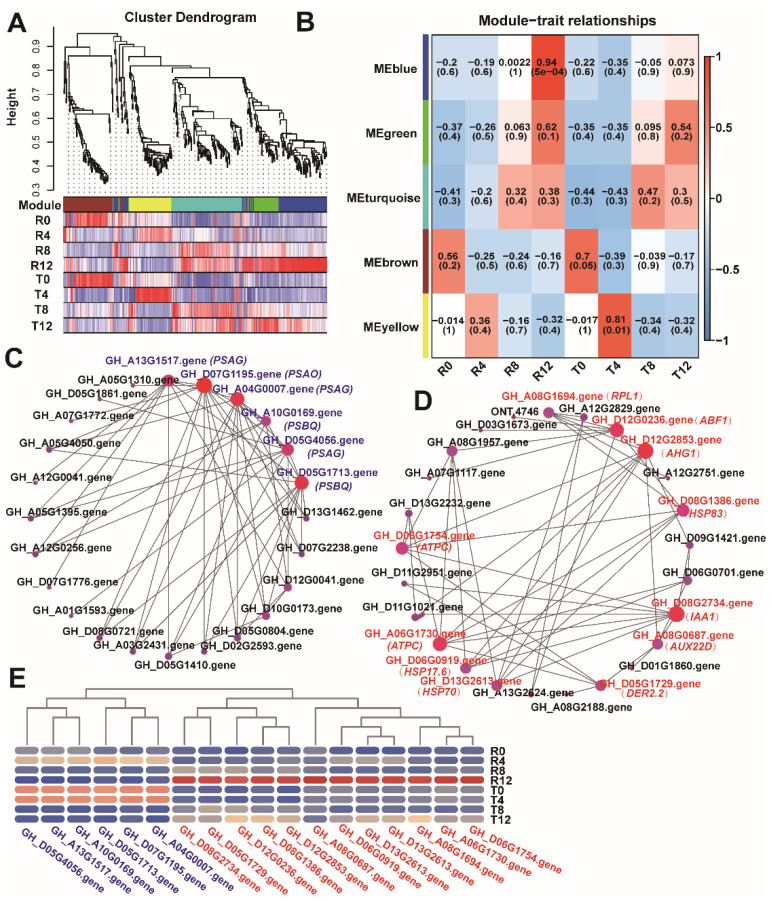
Gene co-expression analysis. (**A**) The cluster dendrogram of all the samples; (**B**) module-trait relationships of the samples; (**C**) network between the genes from the MEyellow module in T4; (**D**) network between the genes from the MEblue module in R12. (**E**) expression profile of the key hub genes.

A total of 1240 DEGs from the screened KEGG pathways (ribosome, plant hormone signaling transduction, and protein processing in endoplasmic reticulum) were used for WGCNA to investigate the gene regulatory network in cotton under high-temperature stress and identify the key genes involved in this biological process. The cluster dendrogram of the eight treatments showed that the branches were mainly classified into five modules (Figure 6A). The module-trait relationship analysis revealed that the R12 and T4 groups were significantly related to the MEblue (*R* = 0.94, *p* < 0.05) and MEyellow modules (*R* = 0.84, *p* < 0.05) (Figure 6B), respectively. Two weighted networks were plotted according to the weighted values between the genes in the MEbule and MEyellow modules to find the potential key genes. In these network diagrams, the larger the size of the nodes, the higher the connectivity of the genes. As shown in Figure 6C, six genes with the highest connectivity were screened as the hub genes from the MEbule module. Among these six hub genes, GH_A13G1517.gene, GH_A04G0007.gene, and GH_D05G4056.gene were annotated as *photosystem I reaction center subunit V* (*PSAG*); GH_D07G1195.gene was annotated as chloroplastic *photosystem I subunit O* (*PSAO*); and GH_A10G0169.gene and GH_D05G1713.gene were annotated as chloroplastic oxygen-evolving enhancer protein 3 (*PSBQ*). The MEyellow module generated 11 hub genes (Figure 6D), namely, one *ribosomal protein* (*50S ribosomal protein L1, RPL1*), three *HSPs* (*HSP70*, *HSP17.6*, and *HSP83*), two auxin signal transduction genes (*IAA1* and *AUX22D*), two ATP synthase gamma chain genes (*ATPC*), one *abscisic acid-insensitive 5-like protein* (*ABF1*), one *protein phosphatase 2C75* (*AHG1*), and one derlin2.2 (*DER2.2*). Expression profiles of the hub genes showed that *PSAG*, *PSBQ*, and *PSAO* had higher expression levels in T0 and T4 than in R0 and R4, whereas, the expression levels of other hub genes were highest in R12 (Figure 6E).

### 2.7. Validation of the Transcriptome Data via RT-qPCR

**Figure 7:**
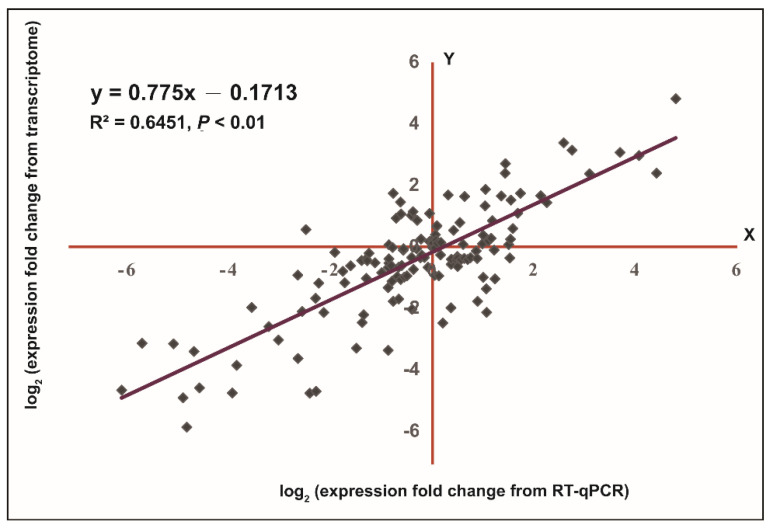
Correlation between the data from the ONT sequencing and RT-qPCR.

Twenty genes were randomly selected for correlation analysis between the ONT transcriptome data and RT-qPCR results to confirm reliability of the transcriptome data. As shown in Figure 7, the data from the ONT transcriptome and RT-qPCR were significantly correlated with R^2^ = 0.6451 (*p* < 0.01), indicating that the ONT transcriptome data were credible in this study.

## 3. Discussion

High-temperature stress is one of the most serious challenges for cotton cultivation because high temperatures often occur during the vegetative and flowering stages of cotton, and causes stunting, poor pollination, and losses of fiber quality in cotton [20]. Breeding heat-tolerant cotton varieties is an effective way to cope with this problem. However, to better understand the heat-tolerant phenomena and produce additional heat-tolerant cotton cultivars, the heat-tolerant mechanism of these variations must be clarified. In this study, ONT sequencing was used to examine the transcription profiles of two distinct heat-responsive cotton cultivars. These findings may reveal the heat-tolerant mechanism of cotton, which needs to be fully elucidated.

Ribosomes are highly conserved ribonucleoprotein complexes that are mainly responsible for protein biosynthesis in organisms [21]. Unlike those of animals or humans, plant cells have three kinds of ribosomes: chloroplast, mitochondrial, and cytoplasmic ribosomes [22]. The cytoplasmic ribosomes are composed of 60S large and 40S small units, whereas the chloroplastic or mitochondrial ribosomes are made up of 50S large and 30S small units [23]. Ribosomal proteins play essential roles in plant growth. In *Arabidopsis thaliana*, several ribosomal proteins have been found to relate to the translation of some specific transcripts and influence some leaf developmental processes [24]. Evidence showed that the mitochondrial ribosomal protein L18 functions in cell division and seed development in *Arabidopsis* [25]. Some other ribosomal proteins were considered to play roles in stress resistance in *Arabidopsis*; for instance, the ribosomal P3 protein of *Arabidopsis* was demonstrated to be an RNA chaperone to enhance the capacity of high-temperature tolerance [26], whereas the plastid ribosomal protein S5 was found to be involved in plant development and low-temperature resistance [27]. Therefore, ribosomal proteins are important in the growth and combating environmental changes of plants. This study revealed that the ribosomal protein genes of the heat-tolerant cultivar responded differently from those of the heat-sensitive cultivar (Figure 2D). Moreover, the expression changes of ribosomal protein mRNAs indicated the widespread and delicate regulation of the biosynthesis of plant protein that promotes the adaptive ability of plants to different environments [28,29]. For example, *RPL1* is a negative translational regulator of some proteins by binding to their mRNAs [30]. This study showed that *RPL1* was one of the hub genes through WGCNA and was most highly expressed in R12 (Figure 6E), which implies that *RPL1* inhibited the generation of some proteins in the sensitive cultivar. These results indicated that they may contribute to the stabilization of protein biosynthesis and the normal growth of the heat-tolerant cultivar cotton plants under long-term high-temperature stress.

HSPs are widely present in organisms and increase in abundance when organisms encounter high temperatures or some other abiotic or biotic stresses [11]. HSPs mainly function as molecular chaperones to promote the folding of newly produced proteins and participate in the refolding and degradation of dysfunctional proteins [31]. In the present study, most of the differentially expressed *HSPs* exhibited remarkable differences in expression level (Figure 3B), indicating that the sensitive cultivar may launch protein processing (refolding or degradation) of dysfunctional proteins more quickly than the tolerant cultivar under heat stress. WGCNA revealed that *HSP83* (GH_D08G1386.gene), *HSP70* (GH_D13G2613.gene), and *HSP17.6* (GH_D06G0919.gene) were the hub genes (Figure 6D). HSP83 responds to heat and protects cells from the heat in Drosophila larvae [32]. HSP70 not only responds to heat but also plays a key role in the degradation of damaged protein and helps to fold the misfolding proteins under stress conditions in *Arabidopsis* [33]. HSP17.6 functions in protein folding and responds to several forms of abiotic stress in *Arabidopsis*, such as salt, osmotic, and heat stresses [34]. Here, these three HSPs were produced more abundantly (Figure 6E) to process a large number of dysfunctional proteins in the sensitive cultivar under high-temperature stress. Thus, the sensitive cultivar may form more dysfunctional proteins than the tolerant cultivar to be processed. As transport protein Sec61 can retrograde transport misfolded proteins out of the endoplasmic reticulum to the cytosol for degradation in yeast [35,36], we suggest that transport protein Sec61 may contribute more to degrade misfolded proteins in the heat-tolerant cultivar than the sensitive cultivar.

This study also revealed that the plant hormone signaling transduction pathway was significantly enriched, and the DEGs in it were mainly involved in the auxin and ethylene-responsive pathways (Figure 3C). A previous study showed that high temperature leads to the differential expression of genes involved in auxin signaling transduction and causes impaired development of cotton anthers [37], indicating that the disordered change in expression levels of genes involved in auxin signaling transduction was not favorable for cotton growth. Here, the expression level of the genes from the auxin signaling transduction pathway changed less dramatically in the tolerant cultivar than in the sensitive cultivar. This phenomenon may protect the heat-tolerant cultivar for normal growth under high-temperature stress. As ethylene-responsive transcription factor 1 participates in the regulation of gene expression remolded by stress factors [38] and promotes germination of lettuce seed under high temperature [39], we suggest that *ethylene-responsive transcription factor 1B-like* may contribute to the thermotolerance of the heat-tolerant cultivar.

High temperature can seriously prohibit photosynthesis in plants by reducing the photosynthetic rate [40] and damaging the thylakoid membrane [41]. These proteins are also light-harvesting complexes and are found in the thylakoid membrane [42]. In *Miscanthus* × *giganteus* treated with chilling, 30 genes associated with chloroplast membrane function, including several *chlorophyll a-b binding proteins*, were found to increase expression to enhance chilling resistance, indicating that the upregulation of *chlorophyll a-b binding proteins* helped protect the photosynthetic system [43]. Here, the expression levels of the *chlorophyll a-b binding proteins* involved in the thylakoid membrane distinctly increased in T4 (Figure 4E), which may imply that they contribute to the maintenance of the thylakoid membrane. Thus, they are conducive to normal photosynthesis in the heat-tolerant cultivar. WGCNA revealed that three photosynthesis-related genes, namely, *PSAG*, *PSBQ*, and *PSAO*, had high expression levels in T0 and T4 (Figure 6C,E). In *Arabidopsis*, *PSAG* plays a vital role in the stability of the photosystem I complex, and lack of *PSAG* leads to sensitivity to photodamage [44]. *PSBQ* is essential for the assembly or stability of photosystem II, and loss of the PSBQ protein causes significant changes in photosystem II function in *Arabidopsis* [45]. *PSAO* involves the balance of excitation energy between photosystems I and II [46]. Taken together, the higher expression of *PSAG*, *PSBQ*, and *PSAO* in the heat-tolerant cultivar than in the heat-sensitive cultivar implied that they contributed to high-temperature resistance.

LncRNAs have long been known to participate in development and stress resistance for plants [47]. For instance, lncRNAs may be involved in ripening and softening in kiwifruit via regulating the expression of genes involved in starch and sucrose metabolism, brassinosteroid biosynthesis, and plant hormone signal transduction [48]. A recent study showed that lncRNAs regulate expression of target genes to enhance high-temperature tolerance in poplar [49]. LncRNAs are also found to play regulatory roles in the stress response in cucumber plants [50]. This study also found that lncRNAs target key candidate genes involved in high-temperature tolerance in cotton, such as *chlorophyll a-b binding proteins*, *ribosomal proteins*, and *heat shock proteins* (Figure 5C). Expression profile analysis of the predicted lncRNAs showed that they had higher expression levels in the heat-tolerant cultivar than in the heat-sensitive cultivar under high-temperature stress. Therefore, lncRNA may play important roles in high-temperature resistance in the heat-tolerant cultivar, but the detailed mechanism still needs to be investigated.

## 4. Materials and Methods

### 4.1. Plant Materials

The two cotton cultivars, namely, ‘Xinluzao36′ and ‘Che61-72′, bred by our laboratory, were heat-tolerant and heat-sensitive types, respectively. The conserved seeds of these two cultivars of cotton were disinfected in 15% hydrogen peroxide solution for 4 h and then rinsed twice with sterile water. The seeds were transferred into geminating boxes (length 12 cm × width 12 cm × height 6 cm) with 1 kg of sterilized sand inside. Ten seeds at equal intervals were sowed in each germinating box, and the sowing depth was 2 cm. After sowing, 300 mL of deionized water was added to the box to saturate the sand with water, and 200 mL water was added to the box every 2 days. The germinating boxes were placed in an illumination incubator (LRH250-G, Hangzhou Deju Instrument, Hangzhou, China). The growth conditions of the seedlings were set as follows: photoperiod, 16 h of day and 8 h of night; temperature, 28 °C during day and 20 °C at night; light density, 300 μmol m^−2^ s^−1^; and relative humidity, 75%. When the cotton seedlings grew into the three-leaf stage, the temperature of the illumination incubator was adjusted to 40 °C for heat stress treatment. The leaf samples were collected at 0, 4, 8, and 12 h after treatment. The samples were designated as T0 (0 h), T4 (4 h), T8 (8 h), and T12 (12 h) for heat-tolerant type ‘Xinluzao36′, and R0 (0 h), R4 (4 h), R8 (8 h), and R12 (12 h) for heat-sensitive type ‘Che61-72′. The samples were frozen in liquid nitrogen and stored at −80 °C. The treatment was conducted three times and all the samples had three biological repetitions.

### 4.2. Library Preparation and ONT Sequencing

RNA for each sample was extracted using the RNAprep Pure Plant Kit (Tiangen, Beijing, China). The concentration and integrity of the RNA were detected to ensure quality for sequencing. cDNA libraries were constructed using 1 μg of RNA from each sample following the method provided by ONT. In brief, full-length mRNA reverse transcription was performed using the SuperScript IV First-Strand Synthesis System (Invitrogen, Carlsbad, CA, USA), followed by cDNA PCR with 14 circles using LongAmp Tag (NEB, Ipswich, MA, USA). The PCR products were then subjected to FFPE DNA repair and end-repair, followed by adaptor ligation using T4 DNA ligase (NEB, Ipswich, MA, USA). DNA purification was conducted using Agencourt XP beads according to the ONT protocol. The final cDNA libraries were added to FLO-MIN109 flowcells and run on the PromethION platform at Biomarker Technology Company (Beijing, China). The eight groups of samples were replicated three times and a total of 24 ONT libraries were generated.

Raw reads were filtered with minimum average read quality score < 7 and minimum read length < 500 bp. Full-length non-chimeric (FLNC) transcripts were determined by searching for primers at both ends of the reads. Clusters of FLNC transcripts were obtained after mapping to the reference genome of cotton published by Hu et al. (2018) [51] by using mimimap2 [52], and consensus isoforms were obtained after polishing within each cluster by pinfish software. Consensus sequences were mapped to the reference genome using minimap2. Mapped reads were further collapsed by the cDNA_Cupcake package, and sequences with coverage <85% and identity <90% were filtered. The non-redundant transcripts were obtained. The ribosomal RNAs also were filtered from the clean reads by matching the reference genome.

The raw sequence data reported in this paper have been deposited in the National Center for Biotechnology Information sequence read archive (Accession No. PRJNA706603).

### 4.3. Functional Annotation of Genes

Coding sequences were predicted by TransDecoder (https://github.com/TransDecoder/TransDecoder (accessed on 12 February 2020)). Functional annotation of genes was performed using the eight databases including the National Center for Biotechnology Information non-redundant protein sequences (Nr), Kyoto Encyclopedia of Genes and Genomes (KEGG), Protein family (Pfam), Eukaryotic Orthology Groups (KOG), Clusters of Orthologous Groups (COG), the evolutionary genealogy of genes: Nonsupervised Orthologous Groups (egg-NOG), Swiss-Prot, and Gene Ontology (GO).

### 4.4. Quantification of Gene Expression Levels and Differential Expression Analysis

Full-length reads were mapped to the reference transcriptome sequence. Reads with match quality above five were further selected for quantification. Expression levels were assessed by reads per gene per 10,000 reads mapped.

Differential expression analysis of two conditions/groups was performed using the DESeq R package (Bioconductor, Buffalo, NY, USA; version 1.18.0). The resulting *p*-values were adjusted using the false discovery rate (FDR) as described by Benjamini and Hochberg [53]. Differentially expressed genes (DEGs) were screened by DESeq with a threshold value of an FDR < 0.01 and foldchange ≥ 2. GO and KEGG functional enrichments of the DEGs were implemented by the GOseq R packages [54] and KOBAS software [55], respectively.

### 4.5. Prediction of lncRNAs and Their Target Genes

LncRNAs do not encode proteins, so they can be predicted by evaluating their encoding potential. This study used CPC [56], CNCI [57], CPAT [58], and Pfam [59] approaches to sort non-protein coding RNA candidates from putative protein-coding RNAs in the transcripts. Transcripts screened from the four approaches were further filtered and a threshold (lengths more than 200 nt and having more than two exons) was applied to select lncRNA candidates. The target genes of the lncRNAs were predicted using the following two methods: (1) based on the correlation of expression levels and the genome position relationship between the lncRNAs and mRNAs; and (2) based on the complementary pairing of lncRNAs and mRNAs. The target genes of the lncRNAs were predicted using the LncTar tool [60]. The overlapping target genes predicted from the two methods were used for analysis.

### 4.6. Weighted Gene Correlation Network Analysis (WGCNA)

The expression values of the screened DEGs above were used for WGCNA via the R package. Modules were obtained using the automatic network construction function blockwiseModules with the default settings. The Pearson correlation coefficient was used to analyze the correlation between each module and the samples. Weight and connectivity of the genes in the module with *p* < 0.05 were further applied to construct a weighted network diagram via the OmicShare tools (Gene Denovo Co. Ltd., Guangzhou, China; http://www.omicshare.com/tools (accessed on 20 August 2021)).

### 4.7. Real-Time Quantitative Polymerase Chain Reaction (RT-qPCR)

To validate the accuracy of gene expression values estimated from the ONT transcriptome, 20 genes from the DEGs with expression levels higher than 10 were randomly selected for RT-qPCR. Primers were designed with Primer Premier 6 (Premier Biosoft, San Francisco, CA, USA) and are listed in Supplementary Table S1. RT-qPCR was conducted with the CFX96 Fluorescent Quantitative PCR System (Bio-Rad, Hercules, CA, USA) and the FastKing One-Step RT-qPCR (SYBR) Kit (Tiangen, Beijing, China). The expression levels were normalized to the internal reference gene nuclear small subunit (SSU) rRNA and calculated by the 2^–ΔΔCt^ method. Three biological and two technical replicates were performed for each sample in RT-qPCR. Results are presented as mean ± standard errors after analysis by one-way ANOVA. Significance was set at *p* < 0.05.5. 

## 5. Conclusions

In this study, one heat-tolerant cotton cultivar and one heat-sensitive cotton cultivar treated with high-temperature stress were chosen for ONT transcriptome analysis. The comparative transcriptome of the two cotton cultivars revealed that the differences in DEG enrichment between them were dominantly concentrated in three KEGG pathways (ribosome, plant hormone signaling transduction, and protein processing in endoplasmic reticulum) and one category of genes (*chlorophyll a-b binding proteins*). The *ribosomal proteins* may contribute more to the stabilization of protein biosynthesis and the normal growth of heat-tolerant cultivar plants under high-temperature stress. *HSPs* and *transport protein Sec61* may enhance the degradation of dysfunction proteins in the heat-tolerant cultivar. Additionally, the auxin and ethylene signaling transduction-related genes responded to high-temperature stress and may be involved in high-temperature resistance. *Chlorophyll a-b binding proteins* may be favorable for photosynthetic homeostasis in the heat-tolerant cultivar. Furthermore, lncRNAs may regulate the expression of these genes to promote the capacity of heat resistance in cotton. This study provides us with insights into the potential heat-tolerant mechanism in cotton.

## Figures and Tables

**Table 1:** Gene annotation statistics.

Annotation Database.	COG	GO	KEGG	KOG	Pfam	Swissprot	eggNOG	Nr	All
Annotated Number	25,469	55,690	26,860	40,413	58,269	53,832	69,684	78,569	78,601

## Data Availability

The raw sequence data reported in this paper have been deposited in the National Center for Biotechnology Information sequence read archive (Accession No. PRJNA706603).

## Supplementary Materials

The following are available online at https://www.mdpi.com/article/10.3390/plants10112517/s1, Figure S1: Annotation analysis of the new genes. Figure S2: Expression heatmap of all the DEGs. Figure S3: KEGG pathway enrichment of the DEGs from the intra-groups of the sensitive and tolerant cotton cultivars. Figure S4: Analysis of the predicted lncRNAs and their target genes. Table S1: Primers for RT-qPCR. Table S2: Statistics of the clean data in each library. Table S3: Full-length transcripts statistics in each sample. Table S4: New gene annotation statistics. Table S5: DEG statistics in each comparison group.
