# Supplementary material for: Nanopore-Based Comparative Transcriptome Analysis Reveals the Potential Mechanism of High-Temperature Tolerance in Cotton (Gossypium hirsutum L.)
Source: Plants (Basel). 2021 Nov 19;10(11):2517. doi: 10.3390/plants10112517 (PMC8618236; doi:10.3390/plants10112517)
Supplement: Supplementary file 1 [file plants-10-02517-s001.zip › plants-1453168-supplementary/Figure S1.pdf]

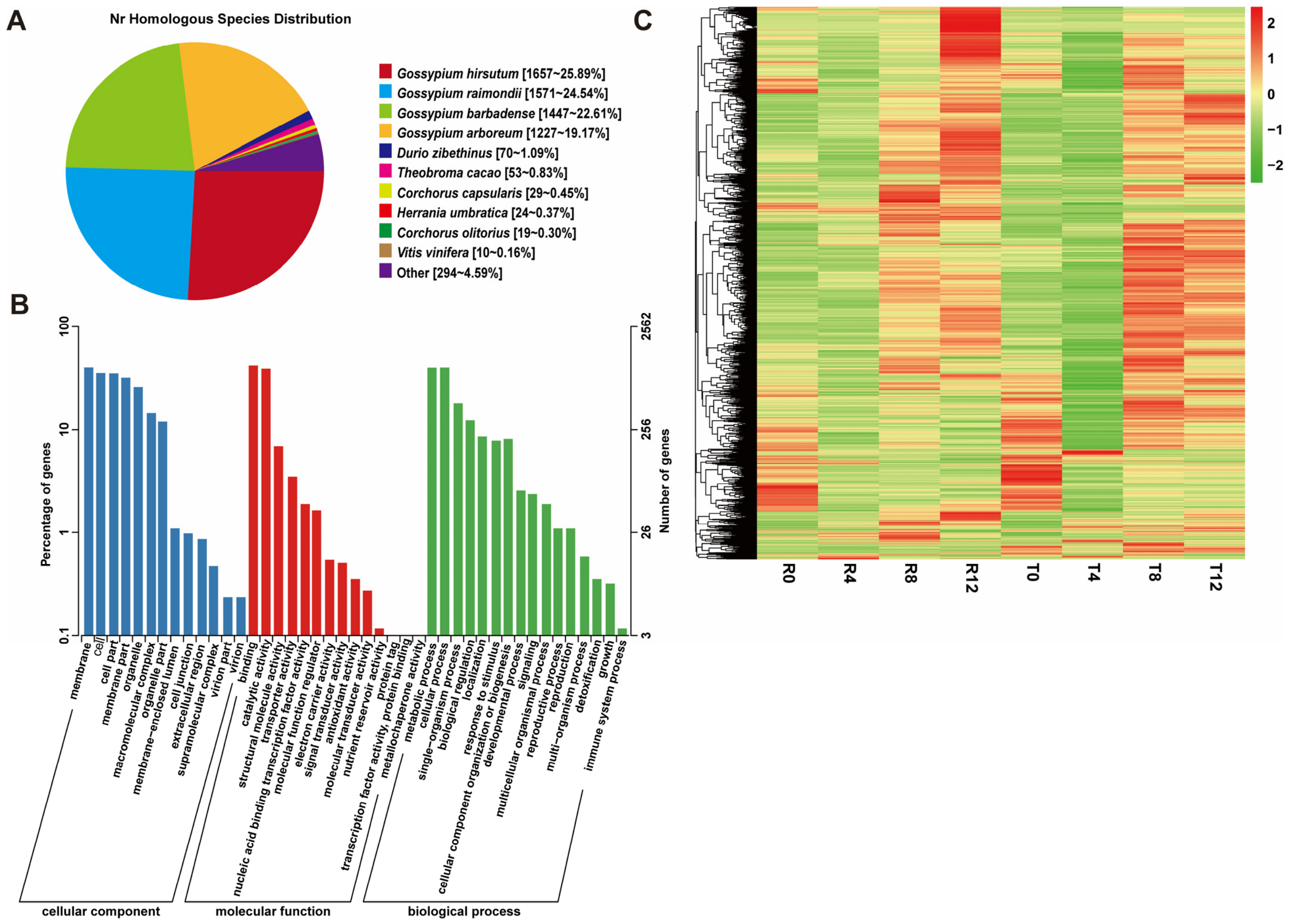


**Figure S1.** Annotation analysis of the new genes. **A:** Homogeneous analysis of the Nr species distribution; **B:** GO term statistics of the annotated genes; **C:** Expression profile of the new genes.
