# Supplementary material for: Nanopore-Based Comparative Transcriptome Analysis Reveals the Potential Mechanism of High-Temperature Tolerance in Cotton (Gossypium hirsutum L.)
Source: Plants (Basel). 2021 Nov 19;10(11):2517. doi: 10.3390/plants10112517 (PMC8618236; doi:10.3390/plants10112517)
Supplement: Supplementary file 1 [file plants-10-02517-s001.zip › plants-1453168-supplementary/Figure S4.pdf]

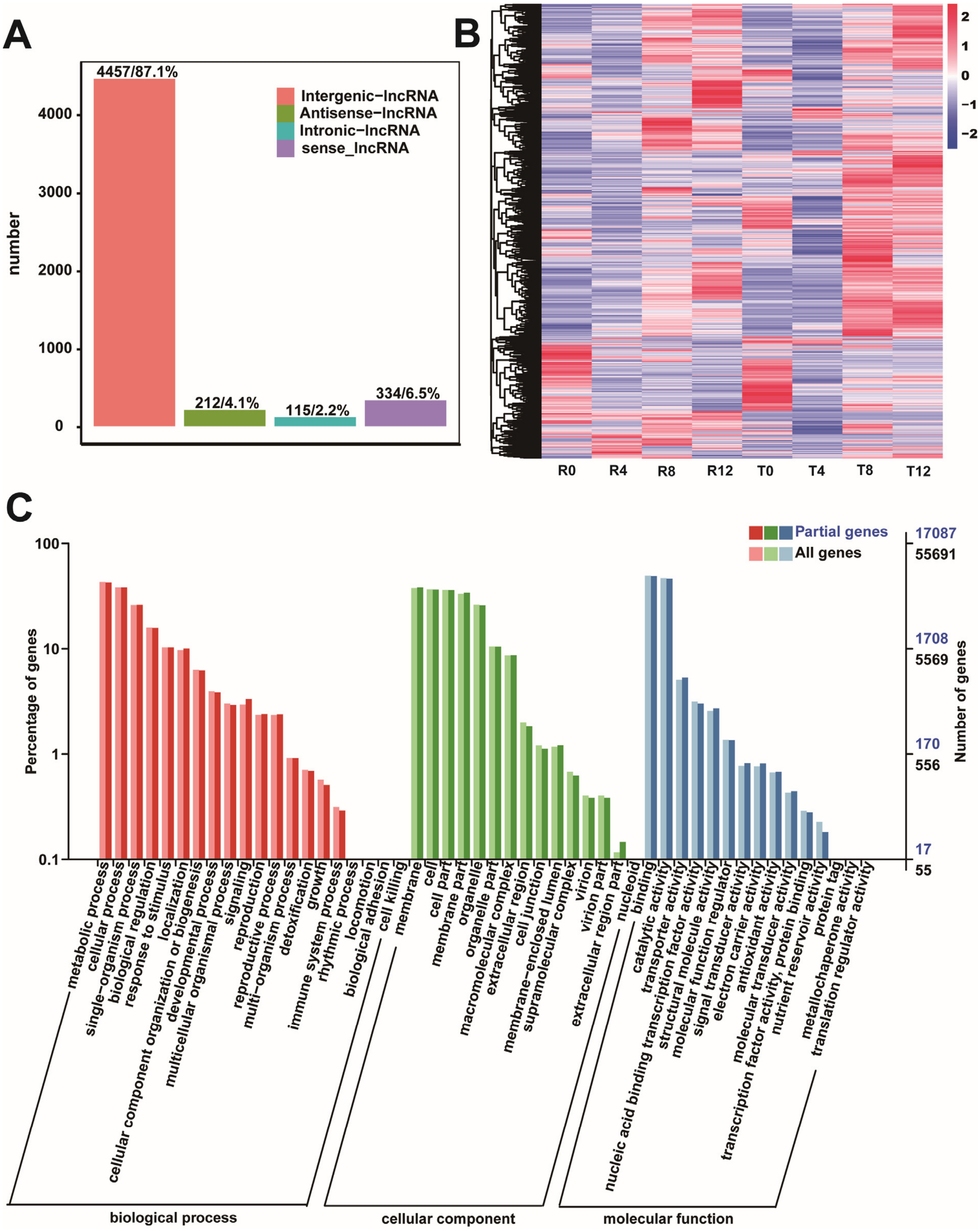


**Figure S4.** Analysis of the predicted lncRNAs and their target genes. **A:** Statistics of the lncRNA types; **B:** Expression profile of the lncRNAs; **C:** GO enrichment of the target genes.
