# Supplementary material for: Nanopore-Based Comparative Transcriptome Analysis Reveals the Potential Mechanism of High-Temperature Tolerance in Cotton (Gossypium hirsutum L.)
Source: Plants (Basel). 2021 Nov 19;10(11):2517. doi: 10.3390/plants10112517 (PMC8618236; doi:10.3390/plants10112517)
Supplement: Supplementary file 1 [file plants-10-02517-s001.zip › plants-1453168-supplementary/Table S1 Primers for RT-qPCR.pdf]

Table S1 Primers for RT-qPCR

Gene	Sequence
GH_A10G1076.gene-F	TGTTCACGGCAGATGATGCT
GH_A10G1076.gene-R	TTTCCCCATCCACGTGCTAC
GH_D10G1805.gene-F	GAAGGGTTGAAGAGCGTGGA
GH_D10G1805.gene-R	AGGAAACGCCGGTAAGAGTC
GH_D01G2035.gene-F	TACTGGTGCTGTGGTTCGTG
GH_D01G2035.gene-R	GCTGCACTTTGCAACTCCTT
GH_A12G2244.gene-F	ATCGAAGTGCTGCCAACTCA
GH_A12G2244.gene-R	TGCCAGGGTAAGAGCAAGTG
GH_A09G0088.gene-F	CGAAAGCATCGGTGGAGAGT
GH_A09G0088.gene-R	TCATCCGACAGTCCAAACCG
GH_D05G0316.gene-F	TTATTGGTTGTGGTCCGGCT
GH_D05G0316.gene-R	ACTATGGTGTCCCGCCAAAC
GH_D08G2157.gene-F	CTGGCTTAGGTTCCGAGTCC
GH_D08G2157.gene-R	ACGCACATAGAATCCGCCAA
GH_D13G1471.gene-F	CTGATTGGAAATTCGCCGGG
GH_D13G1471.gene-R	CCCCACATTCGATGGGCATA
GH_A02G0791.gene-F	AAGCAAGGGTTGCTACTGCT
GH_A02G0791.gene-R	AACCGCGGGTGATATGGTTT
GH_A13G1534.gene-F	ATAAAGCTGGTGCGCGTTTC
GH_A13G1534.gene-R	ACCTCAGGCTCAACAATGGG
GH_A11G1702.gene-F	TCTCATCGGCTACCATTGCC
GH_A11G1702.gene-R	CATGCATTGCACTCTACCGC
GH_D11G1742.gene-F	CGATCTTACACCCGTGCAGT
GH_D11G1742.gene-R	TTCCACATGGTCCAGTAGCG
GH_A03G1577.gene-F	AAGCCACTGACTTTCCCTCG
GH_A03G1577.gene-R	AGCAACCTCCTTCCTAACGC
GH_D01G0967.gene-F	CACCAGGATTCGTGCTGCTA
GH_D01G0967.gene-R	GCCTTGACAACCTGAATGCC
GH_D12G0630.gene-F	GGCCTCAATCGGGCTAGAAA
GH_D12G0630.gene-R	CCGGGGACTATGTTCTGCTC
GH_D02G1748.gene-F	CGTCAGGTGGTTGCTTTGTG
GH_D02G1748.gene-R	AGGATACCCATCACGCCTCT
GH_A12G0256.gene-F	TGAGTGCCCAGATGACGTTT
GH_A12G0256.gene-R	AAACCAGCGGCAATTTGGTC
GH_D12G0262.gene-F	AAGCTGGTGACTCCTGATGG
GH_D12G0262.gene-R	ATCCGACTGATCCACACTGC
GH_D07G1195.gene-F	TCATCCCCACCGAACAACAC
GH_D07G1195.gene-R	CAATCCATCCGATCAGCCCA
GH_A12G1558.gene-F	AACCCCAACTTCCAAGCTCAA
GH_A12G1558.gene-R	AACCCCTCCAAGAGCAAGAC
GH_A06G1891.gene-F	GCGTCACATTTCTTCGGGAC
GH_A06G1891.gene-R	TCGCATTGGGGAACCAAACA
GH_A04G0248.gene-F	TTGGGACACTGCTGGTTTGT
GH_A04G0248.gene-R	GAGCTCCTGCCTTGAACCAT
GH-SSU-F	AACTTAAAGGAATTGACGGAAG
GH-SSU-R	GCATCACAGACCTGTTATTGCC
