# Supplementary material for: Nanopore-Based Comparative Transcriptome Analysis Reveals the Potential Mechanism of High-Temperature Tolerance in Cotton (Gossypium hirsutum L.)
Source: Plants (Basel). 2021 Nov 19;10(11):2517. doi: 10.3390/plants10112517 (PMC8618236; doi:10.3390/plants10112517)
Supplement: Supplementary file 1 [file plants-10-02517-s001.zip › plants-1453168-supplementary/Table S2 Statistics of the clean data.pdf]

Table S2. Statistics of the clean data in each library

Sample	N50 Length	Mean Length	Max Length	Mean Q score
R0-r1	1001	943	11965	11
R0-r2	1000	948	12735	11
R0-r3	981	922	12006	11
R12-r1	1153	1052	31126	11
R12-r2	1081	1000	12235	11
R12-r3	1153	1055	16209	11
R4-r1	1031	991	13626	11
R4-r2	986	927	12164	11
R4-r3	1015	968	17650	11
R8-r1	1070	1005	12204	11
R8-r2	1131	1057	14250	11
R8-r3	1130	1053	12178	11
T0-r1	939	883	29910	11
T0-r2	927	882	53252	11
T0-r3	943	888	28140	11
T12-r1	992	943	10939	11
T12-r2	1037	972	10084	11
T12-r3	987	940	9406	11
T4-r1	967	914	46105	10
T4-r2	962	911	32915	11
T4-r3	1012	961	10978	11
T8-r1	1040	973	12100	11
T8-r2	1039	975	10385	11
T8-r3	1003	950	12219	11
