# Supplementary material for: Nanopore-Based Comparative Transcriptome Analysis Reveals the Potential Mechanism of High-Temperature Tolerance in Cotton (Gossypium hirsutum L.)
Source: Plants (Basel). 2021 Nov 19;10(11):2517. doi: 10.3390/plants10112517 (PMC8618236; doi:10.3390/plants10112517)
Supplement: Supplementary file 1 [file plants-10-02517-s001.zip › plants-1453168-supplementary/Table S3 Full-length transcripts statistics.pdf]

Table S3. Full-length transcripts statistics in each sample.

Sample	Number of clean reads	Number of full-length reads	Full-Length Percentage (%)
R0-r1	7,458,544	5,796,611	77.72%
R0-r2	8,784,146	6,743,250	76.77%
R0-r3	8,806,212	6,761,816	76.78%
R12-r1	9,406,255	7,545,051	80.21%
R12-r2	8,998,475	7,269,454	80.79%
R12-r3	8,160,985	6,642,112	81.39%
R4-r1	7,190,038	5,450,625	75.81%
R4-r2	8,062,262	6,380,621	79.14%
R4-r3	7,413,865	5,693,959	76.80%
R8-r1	8,229,395	6,528,872	79.34%
R8-r2	7,357,241	5,690,484	77.35%
R8-r3	7,961,439	6,296,224	79.08%
T0-r1	7,953,896	6,384,761	80.27%
T0-r2	8,311,046	6,507,387	78.30%
T0-r3	8,035,449	6,327,212	78.74%
T12-r1	7,127,052	5,744,429	80.60%
T12-r2	6,509,499	5,088,906	78.18%
T12-r3	7,180,722	5,731,610	79.82%
T4-r1	7,791,760	6,027,909	77.36%
T4-r2	8,212,003	6,362,641	77.48%
T4-r3	7,792,607	6,161,558	79.07%
T8-r1	6,799,663	5,497,080	80.84%
T8-r2	6,994,128	5,698,753	81.48%
T8-r3	6,992,522	5,582,299	79.83%
