# Supplementary material for: Nanopore-Based Comparative Transcriptome Analysis Reveals the Potential Mechanism of High-Temperature Tolerance in Cotton (Gossypium hirsutum L.)
Source: Plants (Basel). 2021 Nov 19;10(11):2517. doi: 10.3390/plants10112517 (PMC8618236; doi:10.3390/plants10112517)
Supplement: Supplementary file 1 [file plants-10-02517-s001.zip › plants-1453168-supplementary/Table S4 New gene annotation statistics.pdf]

Table S4 New gene annotation statistics

Annotation Database	COG	GO	KEGG	KOG	Pfam	eggNOG	Swissprot	Nr	All
Annotated Number	750	2,562	1,536	2,494	1,167	3,830	2,588	6,402	6,415
