# Supplementary material for: Nanopore-Based Comparative Transcriptome Analysis Reveals the Potential Mechanism of High-Temperature Tolerance in Cotton (Gossypium hirsutum L.)
Source: Plants (Basel). 2021 Nov 19;10(11):2517. doi: 10.3390/plants10112517 (PMC8618236; doi:10.3390/plants10112517)
Supplement: Supplementary file 1 [file plants-10-02517-s001.zip › plants-1453168-supplementary/Table S5 DEG statistics in each comparison group.pdf]

Table S5 DEG statistics in each comparison group

DEG set	DEG Number	up-regulated	down-regulated
**R0_vs_R12**	8,870	4,369	4,501
**R0_vs_R4**	2,764	1,671	1,093
**R0_vs_R8**	5,179	2,302	2,877
**R0_vs_T0**	365	174	191
**R12_vs_T12**	3,201	1,355	1,846
**R4_vs_R8**	1,438	385	1,053
**R4_vs_T4**	2,303	996	1,307
**R8_vs_R12**	3,520	2,280	1,240
**R8_vs_T8**	1,339	705	634
**T0_vs_T12**	6,480	3,597	2,883
**T0_vs_T4**	4,582	2,527	2,055
**T0_vs_T8**	4,260	1,821	2,439
**T4_vs_T8**	6,521	3,030	3,491
**T8_vs_T12**	927	639	288
**Total**	19,600	--	--
