# Supplementary figures and images for: Nanopore-Based Comparative Transcriptome Analysis Reveals the Potential Mechanism of High-Temperature Tolerance in Cotton (Gossypium hirsutum L.)
Source: Plants (Basel). 2021 Nov 19;10(11):2517. doi: 10.3390/plants10112517 (PMC8618236; doi:10.3390/plants10112517)

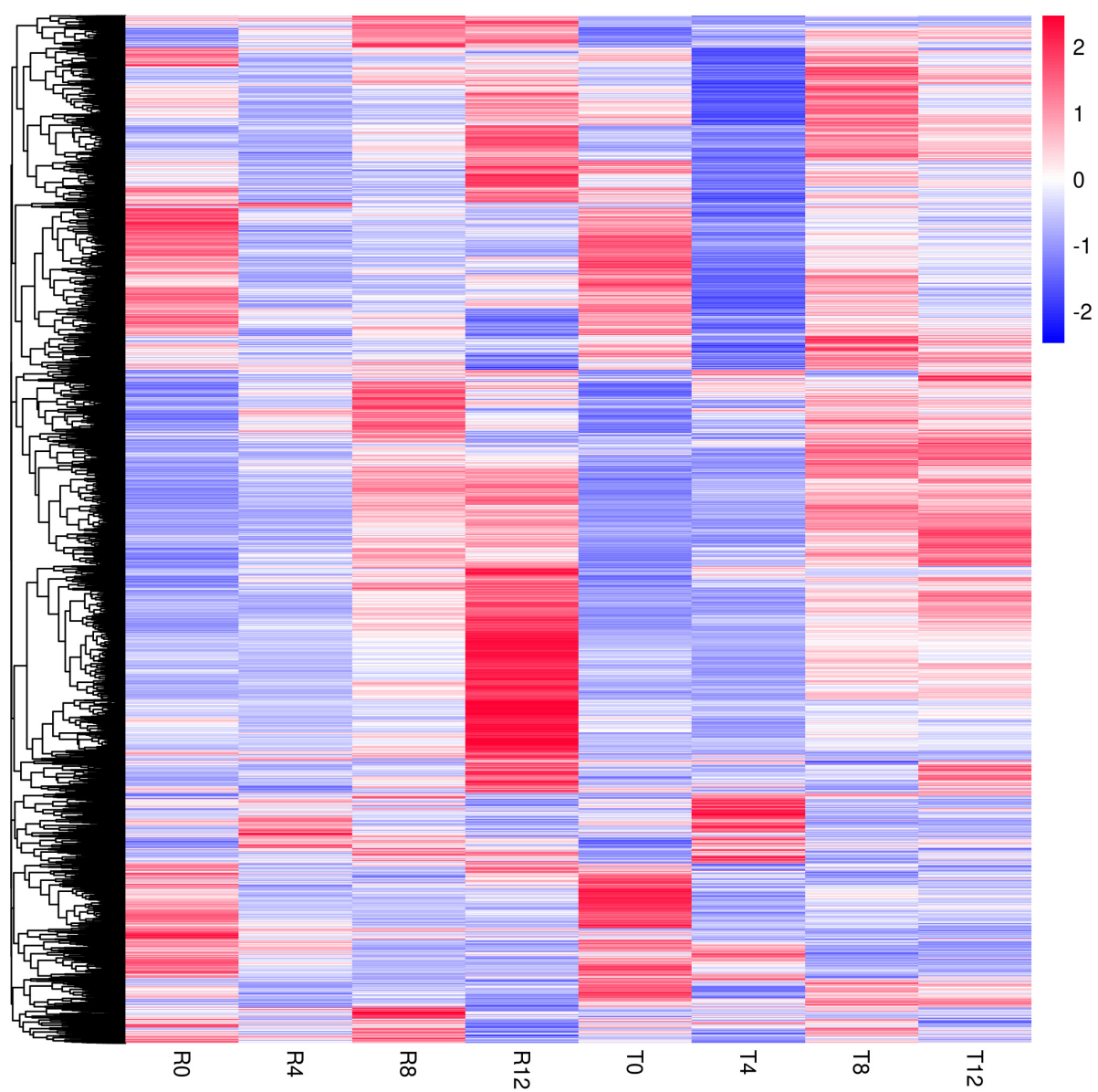

**Figure S2.** Expression heatmap of all the DEGs.

Supplement: Supplementary file 1 [file plants-10-02517-s001.zip › plants-1453168-supplementary/Figure S2.pdf]
